# Why Systems Thinking Is Needed to Center Trust in Health Policy and Systems: Comment on "Placing Trust at the Heart of Health Policy and Systems"

**DOI:** 10.34172/ijhpm.8706

**Published:** 2024-09-18

**Authors:** Diane T. Finegood, Chris Yakimov

**Affiliations:** ^1^Morris J. Wosk Centre for Dialogue and Department Kinesiology and Biomedical Physiology, Simon Fraser University, Vancouver, BC, Canada.; ^2^Faculty of Education, Simon Fraser University, Vancouver, BC, Canada.

**Keywords:** Trust, Systems Thinking, Complexity, Healthcare, Organizational Change

## Abstract

The editorial by McKee and colleagues is an important call to action to put a spotlight on trust and its role in the function of health systems. The authors make a good case for this focus considering how trust in health systems seems to have eroded in recent years, an erosion accelerated by the COVID-19 pandemic. They recognize that trust is complex given the many forms of trust, the importance of context, and its dynamic and unpredictable nature. However, the solutions they offer including learning how to measure trust and figuring out the causes and consequences of trust are just simple or complicated solutions to this complex challenge. Instead, we need to approach building trust in healthcare by embracing and harnessing complexity. This starts by understanding the difference between complicated and complex challenges and then by applying complex systems frameworks that offer insight into new ways forward.

 Everyone intuitively knows trust is important in virtually every interaction between people and organizations. In the healthcare system, this includes patients, providers and decision-makers, as well as clinics, hospitals and governments. Some of what makes trust complex is its embodiment in day-to-day behaviour, its unpredictability, its interdependence with fear, and the fact that it is something we practice individually. Trust is a matter of reciprocal relationships and commitments.^1^

 McKee and colleagues acknowledge that trust is not just one thing and that different forms of trust as described by Solomon and Flores are at play in the healthcare system.^2,3^ Simple trust—unthinking, naive trust based on familiarity, that is transparent and taken for granted—can be found throughout the system, such as in the faith some people have that their doctor will always make the right diagnosis.

 Basic trust—the ability and willingness to meet people without inordinate suspicion—is needed in the relationship between health workers and their employers, as suggested by McKee et al, but also between patients and their providers, and between citizens and their governments. Where trust in government systems was high during the COVID-19 pandemic, there was greater compliance with precautionary measures.^4^ Basic trust in providers and health systems is often lacking in people from communities that have historically been mistreated and continue to experience racism in healthcare settings.^5^

 Authentic trust—trust that is mature, articulated and carefully considered—is what many hope for in their relationship with their healthcare providers. Authentic trust requires consideration of what it takes to create, maintain and restore relationships. A majority of adults in Canada rank “knowing me as a person,” “stand up for me,” and “help me to reach my health goals” as fairly to very important.^6^ Systems where engagement with your provider is limited to only a few minutes do not support building authentic trust—they demand simple or basic trust. Unfortunately, these types of trust are difficult to repair when lost, whereas authentic trust strengthens communication and enables better decision-making within the complexity of the systems at play that influence or determine an individual’s health.

 Authentic trust is also needed in the relationship between healthcare providers and the organizations they work for. Current barriers to building trust in these relationships include the command-and-control structures of these organizations and the deeply embedded cultures of fear and risk avoidance.Fear of failure and punishment places an inordinate emphasis on success and a desperate avoidance of risk. These cultures and beliefs give us systems that try to control risk and prevent failure. Unfortunately, the policies and procedures that impose control tend not to support the collaboration and teamwork that build trust.

 In systems driven by fear and the perceived need for control, another form of trust takes hold, cordial hypocrisy—pretend trust, a façade of trust that hides cynicism and distrust. When we pretend that we trust, we withhold critical information and shut down communication, the opposite of what is needed in complexity and in healthcare.^3^ Institutions often encourage cordial hypocrisy in the name of minimizing friction, but the result is just the opposite, institutionalization of distrust.

## Complex Is Not the Same as Complicated

 McKee and colleagues suggest “this situation cannot continue” and that a key priority should be to measure trust, despite the lack of standardized measures and potential cost.^2^ Their purpose in focusing on measuring trust is to “help understand how trust is eroded and how it can be built, restored, and protected.” They acknowledge that measurement is not enough and will need to be translated into effective actions that build trust over time.

 This approach might be appropriate for a problem that is just simple or complicated, but not one that is complex. Complicated systems are mostly predictable, controllable and/or designable, while complex systems are often unpredictable, self-organizing and/or emergent.^7^ The notion that measurement can lead to understanding which enables predictable results and “best” or “good” practices makes sense for simple or complicated problems. But in complexity the relationship between cause and effect can only be understood in hindsight and the unpredictable and self-organizing nature of complex systems makes this strategy less helpful and delays the development of more effective strategies.^6^

 It is false to assume that working out the causes of trust and distrust will provide necessary evidence that leads to hypotheses on which we can test approaches to building trust. Adam and Donelson suggest that rather than traditional variance models with trust or distrust as an outcome, we need more process models that focus on the evolution of relationships and state changes as mediated by events, activities and choices.^1^ Rather than focusing on measures of trust as an outcome, we need to focus on the factors that build reciprocity like fulfillment of self-interest, common goals, gratitude and shared power, responsibility, and authority.

 Robinson and Simard point out “problem-oriented” research is responsible for leaving many important applied research questions unanswered and for slowing progress on complex challenges like childhood obesity.^8^ Instead, they suggest we need more “solution-oriented” research which emphasizes the identification of improved health. They also suggest we need to focus on research questions that would change how we approach clinical, policy and public health problems. Solution-orientation is consistent with a collaborative, continuous learning approach.

 As Rittel and Weber point out, “there is no definitive formulation of a wicked problem” and “every wicked problem is essentially unique.”^9^ The diversity of types of trust and the individual nature of the practice of building trust make interpretation of trust measures difficult. The law of requisite variety suggests we will need as many responses as the diversity of the conditions for building trust. Trust needs to be understood at and by the individual who is building trust in a particular context and moment in time.

 Instead of thinking that solutions for complicated problems apply in complexity, we must deepen our understanding of the different nature of complex problems. Glouberman and Zimmerman explained the difference between simple, complicated and complex challenges with the metaphors of baking a cake, sending a rocket to the moon and raising a child, respectively.^10^ When baking a cake, the problem is clear, the same rules apply every time, and the outcome is standardized. You can have a “best” practice or recipe for success. When sending a rocket to the moon there is some uncertainty, trial and error is helpful and each effort can improve the success of subsequent efforts. Expertise is needed and you can have a “good” practice, an approach that has a high probability of success. But in raising a child there is often uncertainty, each child and their relationships and context are unique and while rules may be helpful, they can also make things worse. In complexity, practice is “emergent.” No one process, like raising a child, is only complex. There are many elements that may be simple or complicated, but in complexity the whole is more than the sum of the parts making predictability difficult.

 Stacey described simple, complicated and complex in terms of levels of agreement and levels of certainty.^7^ When agreement and certainty about what to do are high, then the challenge is relatively simple. As agreement and/or certainty erode, a problem becomes more complicated and when one or both are at a minimum the situation becomes complex or even chaotic. Clearly trust is interdependent with perceived levels of certainty and agreement. As certainty and agreement decrease, complexity increases, and building trust becomes increasingly important. Low trust environments make it difficult to build the reciprocal relationships needed to function in complex systems. In high trust environments, complexity decreases.^3^

## Applying Complex Systems Frameworks

 So how can we shape what emerges from the complexity of our healthcare systems? How can we centre trust and build in more trust-based approaches?

 Application of frameworks that describe the differences between simple, complicated and complex also hint at how we may need to approach the development of strategies to build more trust in healthcare. Glouberman and Zimmerman point out that when every situation is unique, they each provide experience, but learning and adaptation is key.^10^ Expertise can be helpful, but it may not be necessary, and rules may be helpful but can also make things worse. Principles that allow for adaptation to the context and circumstances are more helpful than rules.^7^

 Instead of trying to work out the causes of trust and distrust using linear models, we should unpack the complexity of trust using frameworks like Meadows’ Places to Intervene or the more condensed Intervention Level Framework.^7^ These frameworks speak to the importance of our deeply held beliefs and how they drive the systems and structures we create. Control and risk avoidance tend to create an accountability paradigm, one that emphasizes targets, requires independent assessment and punishes failure. In complexity, learning needs to be prioritized, flexibility and adaptability are needed, and failure embraced. For example, the culture of blame in healthcare has a negative effect on reporting of adverse events because healthcare workers fear they will be punished.^11^ The measurements needed to create accountability (eg, frequency of adverse events) are different from those required for learning and process improvement (eg, near miss reporting).

 Another paradigm shift that would help build trust is if we understood healthcare as a relational practice rather than a transactional one.^12^ So much of healthcare is transactional. It is broken down into discrete exchanges that are formalized in contracts, rules and practice guidelines. The systems that track transactions are often outdated and require considerable resources. It is hard to have a long-term focus when transactions drive costs and allocation of resources often depends on political decision-making. Building trust in a transactional environment is challenging, especially given the complexity of delivering healthcare in a universal and equitable fashion.

 If we could shift from viewing healthcare as transactional to a more relational approach, then we could focus on building relationships and creating a foundation for longer-term perspectives.^12^ A relational approach requires dialogue and time to support the building of authentic trust. Many systems frameworks illustrate the spectrum of trust and the types of actions that help to build trust.^7^ The Collaboration Spectrum illustrates the spectrum from competition or co-existence, to communication and cooperation, through to coordination, collaboration and integration. Trust necessarily increases as individuals and organizations in relation to each other move towards collaboration and integration. As the trust needed to build collaboration increases, “turf” necessarily decreases.^7^

 Wheatley and Frieze suggest that process matters when we want to use the concept of emergence to make change at scale.^7^ In their two-loop model, trust can be built as innovators come together and create connections. The model acknowledges the importance of letting people come together to meet their own needs first, not in some form of coerced connection. As their needs get met through interacting with others, the foundation for ongoing connection begins to build, similar to the process model of Adam and Donelson^1^. If our systems then support and nourish these connections by providing resources to build communities of practice, then systems of influence can arise. Communities of practice are structures where individuals come together for a common theme or purpose. They build trust among members, share tacit knowledge and develop shared practice and collective intelligence. This adds to the implicitly held knowledge by the individuals in the group and creates the conditions for a system of influence to emerge.^7^

 Bar-Yam et al suggest we can incentivize collaboration by recognizing the interdependence of competition and collaboration.^13^ By empowering competition between work groups, groups of care providers can be responsible for medical outcomes and performance metrics. For care to improve, the people engaged in providing care, who know the most about what to do, need to be collectively responsible for outcomes. Workgroup performance, which depends on things like communication, relational capacity and trust, needs to be measured at the level of the group not the individual because outcomes often rely on an entire group’s performance. An accountability frame in a hierarchy for individuals is toxic to environments where learning partnerships and trust building need to be nurtured. Teams where the members are collaborative and trust is high, have better outcomes to contribute to the success of the healthcare system as a whole.

 French and colleagues have introduced the complexity theory of outcome creation (CTOC) which recognizes that individual outcomes are the result of complex interaction with systems. CTOC is in opposition to the rationalist theory which often presumes a simple and direct causality between services and outcomes.^14^ CTOC suggests that learning partnerships, like collaborative workgroups, are needed to steward systems toward creativity and resilience, strengthen coordination to align stakeholders and resources and to enable adaptation in response to changing conditions. Learning partnerships can also benefit from the process of building a shared measurement system.^15^

## A Paradigm Shift for the Future

 Many have come to recognize the complexity of our healthcare systems, and clearly building trust is needed to improve system function and the experience and outcomes for individuals who are part of or engaged with these systems. We need to embrace and harness this complexity rather than think we can work out all the important causal relationships and find solutions in these linkages.

 Here we illustrate just a few of the many different frameworks developed by systems thinkers.^7^ These frameworks offer suggestions like the need to work at shifting the paradigm from transactional to relational and from accountability to learning by focusing on principles rather than rules. Instead of investing in working out the causes of trust and distrust we should invest in networks, workgroups and communities of practice that focus on sharing knowledge, building trust and building shared practice. We should build learning systems that embrace failure and use metrics that enhance the learning process. Learning partnerships can help steward, coordinate and adapt action in real time supported by shared measurement systems. To walk the talk of a complex systems approach, we need to recognize building trust is not an outcome, but an ongoing process.

## Ethical issues

 Not applicable.

## Conflicts of interest

 Authors declare that they have no conflicts of interest.
